# The Atlanta Medical and Surgical Journal

**Published:** 1881-09-20

**Authors:** 


The Atlanta Medical and Surgical Journal, formerly the organ of the Atlanta Medical College, has made a new departure, and is now issued as an Independent Journal under the name of *The Atlanta Medical Register.* It is edited by Dr. Thad Johnson, Professor of Surgery in The Southern Medical College, and Dr. J. B. Baird, of this city.

